# A Bioorthogonal Probe for Multiscale Imaging by ^19^F-MRI and Raman Microscopy: From Whole Body to Single Cells

**DOI:** 10.1021/jacs.1c05250

**Published:** 2021-07-28

**Authors:** Cristina Chirizzi, Carlo Morasso, Alessandro Aldo Caldarone, Matteo Tommasini, Fabio Corsi, Linda Chaabane, Renzo Vanna, Francesca Baldelli Bombelli, Pierangelo Metrangolo

**Affiliations:** † Laboratory of Supramolecular and Bio-Nanomaterials (SupraBioNanoLab), Department of Chemistry, Materials, and Chemical Engineering “Giulio Natta”, Politecnico di Milano, Via Luigi Mancinelli 7, 20131 Milan, Italy; ‡ Istituti Clinici Scientifici Maugeri IRCCS, Via S. Maugeri 4, 27100 Pavia, Italy; § Department of Chemistry, Materials, and Chemical Engineering “Giulio Natta”, Politecnico di Milano, Via Luigi Mancinelli 7, 20131 Milan, Italy; ∥ Department of Biomedical and Clinical Sciences “Luigi Sacco”, Università di Milano, Via G. B. Grassi 74, 20157 Milan, Italy; ⊥ Experimental Neurology (INSPE) and Experimental Imaging Center (CIS), Neuroscience Division, IRCCS Ospedale San Raffaele, Via Olgettina 60, 20132 Milan, Italy; ¶ CNR-Institute for Photonics and Nanotechnologies (IFN-CNR), Department of Physics, Politecnico di Milano, Piazza Leonardo Da Vinci 32, 20133 Milan, Italy

## Abstract

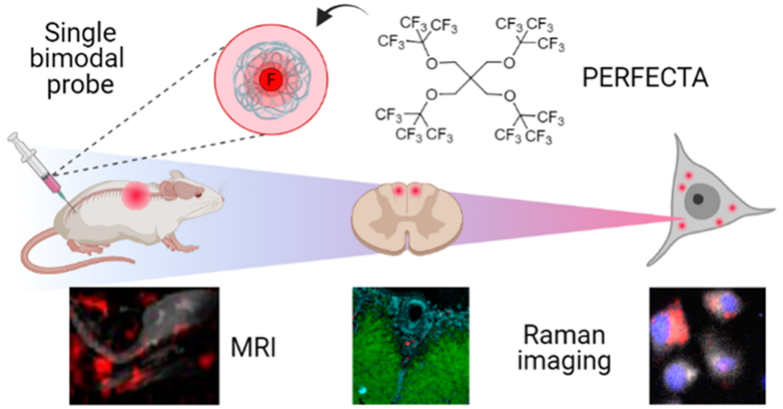

Molecular imaging techniques are essential tools for better investigating biological processes and detecting disease biomarkers with improvement of both diagnosis and therapy monitoring. Often, a single imaging technique is not sufficient to obtain comprehensive information at different levels. Multimodal diagnostic probes are key tools to enable imaging across multiple scales. The direct registration of *in vivo* imaging markers with *ex vivo* imaging at the cellular level with a single probe is still challenging. Fluorinated (^19^F) probes have been increasingly showing promising potentialities for *in vivo* cell tracking by ^19^F-MRI. Here we present the unique features of a bioorthogonal ^19^F-probe that enables direct signal correlation of MRI with Raman imaging. In particular, we reveal the ability of PERFECTA, a superfluorinated molecule, to exhibit a remarkable intense Raman signal distinct from cell and tissue fingerprints. Therefore, PERFECTA combines in a single molecule excellent characteristics for both macroscopic *in vivo*^19^F-MRI, across the whole body, and microscopic imaging at tissue and cellular levels by Raman imaging.

## Introduction

A pressing need in modern medicine is the development of ever more sensitive imaging tools to improve early diagnosis and support personalized medicine and precision surgery.^1^ In the past decade, multimodal imaging enabled detailed examination of human biology at multiscales, from structural anatomical characteristics to physiopathological features at the molecular level. With the increasing request of correlation and integration of various imaging information at different scales, the development of multimodal probes is highly needed.^2^

**Figure 1:**
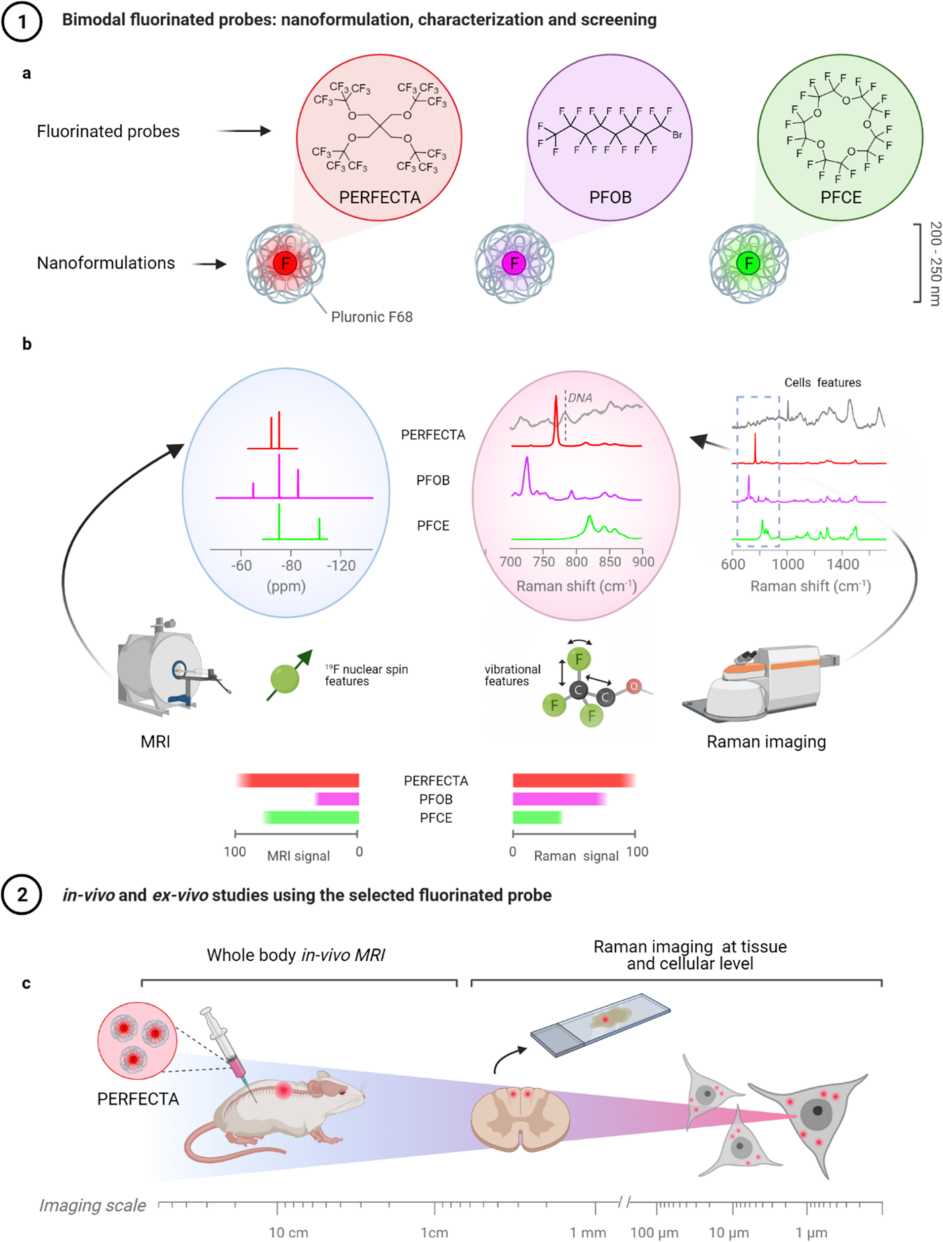
Schematic representation of fluorinated probe nanoformulations and bimodal imaging strategy. (a) The three fluorinated probes (i.e., PERFECTA, perfluorooctylbromide named as PFOB, and perfluoro-15-crown-5-ether shortened as PFCE) were first nanoformulated as an emulsion of a nonionic surfactant (Pluronic F68) to obtain fluorinated probe-loaded NPs. (b) Nanofluorinated probes were studied by both ^19^F-NMR and Raman imaging to identify specific spectral features and characterize their performance, including signal intensity and specificity. (c) After selecting PERFECTA as the best bimodal imaging probe, *in vivo* experiments were performed to show the advantage of this new bimodal imaging approach at multiscales from whole body (i.e., centimeter size) to histological and subcellular size (i.e., micrometer size).

Raman spectroscopy (RS) is a direct and nondestructive approach based on the highly specific recognition of chemical bonds present in the analyzed sample. RS has been proved to be crucial in studying biological samples and has major potential for clinical translation.^3,4^ Even if Raman microscopy is largely used as a label-free technique, enabling distinction between healthy and pathological cells or tissues bypassing the use of fluorescent markers, specific Raman probes may further extend sensitivity and specificity. These probes are typically based on small chemical groups with diagnostic vibrational features, such as alkynes^5^ and deuterated compounds.^6^ Alternatively, plasmonic nanoparticles (NPs) were studied as SERS (surface-enhanced Raman scattering) tags.^7^ All these probes have been widely used for imaging biological targets in cells and tissues.^8,9^ While Raman probes paved the way toward super-multiplexed optical imaging^10^ and bioorthogonal cell- or organelle-targeted imaging, in most cases they are not directly detectable by clinical imaging modalities (i.e., MRI). An exception is represented by some SERS tags, based on the synthesis of multimetal NPs, which have been proposed as bimodal agents, but are often limited by toxicity and stability issues.^11,12^ For this reason, new biocompatible and bimodal probes are essential to allow the integration of imaging analysis of structural and chemical biology with *in vivo* functional imaging. Here we demonstrate, for the first time, the ability of fluorinated molecules to work as bimodal probes (Figure 1), allowing the combination of *in vivo*^19^fluorine-magnetic resonance imaging (^19^F-MRI), used to explore whole body or organs (i.e., with a spatial resolution from 40 μm to 2 mm), with Raman microscopy, enabling tissue and cell imaging with subcellular resolution (∼500 nm) and high biomolecular specificity.^3^

^19^F-MRI is indeed emerging as a powerful noninvasive imaging technique, easily translatable for clinical use,^13,14^ as it permits in-depth *in vivo* quantification without the use of radioactive agents and can be combined with standard ^1^H-MRI protocols. Moreover, lack of endogenous organic ^19^F atoms in living tissues permits unambiguous *in vivo* visualization of exogenous fluorinated probes as colored “hot spots” on the anatomical images obtained by multiparametric ^1^H-MRI.^13−15^ Until now, ^19^F-MRI has been combined with fluorescence imaging,^16^ enabling analysis at the cellular level. However, this approach has often been hampered by the use of two distinct chemical agents, which are commonly co-incorporated in nanoformulations.^17,18^ Moreover, the fluorescent signal is unstable over time, is easily quenched, and could be dissociated from the fluorinated probe as the nanoformulation is degraded.^19^ Instead, direct covalent functionalization of the fluorinated probe with a fluorescent tag may alter its physicochemical and pharmacokinetic properties.^20^ Compared to fluorescence imaging, Raman probes are generally smaller, stable, and unaffected by quenching processes and may guarantee higher multiplexing capabilities due to very narrow vibrational signals, permitting over 20-color channels with Raman imaging.^8,21^ On the other hand, Raman is mainly limited by the intrinsically low cross-section of the Raman effect, resulting in weak signals and low imaging speed, if compared to fluorescence-based approaches. In this context, recently developed imaging enhancing strategies, such as stimulated Raman scattering (SRS), permitted to accelerate imaging performance close to a fluorescent-based one.^9,22^ The strength of our approach resides in the combination of Raman microscopy and ^19^F-MRI for bioimaging at multiscales with high versatility and specificity by using a single chemical imaging agent (Figure 1). Moreover, fluorinated probes can be considered twofold bioorthogonal thanks to their chemical and biological inertness, due to strong C–F bonds, and total absence of endogenous organic fluorine.^23^ In this work, the characterization of three fluorinated probes, commonly used for ^19^F-MRI, in terms of their magnetic and vibrational features is presented. This screening showed that the unique chemical features of a superfluorinated probe, PERFECTA,^24,25^ a highly symmetric branched molecule with 36 magnetically equivalent ^19^F atoms as Csp^3^–F bonds, were superior in both imaging modalities with a sharp ^19^F-NMR signal^26^ in addition to a strong and distinctive Raman signature. Finally, the use of PERFECTA as an *in vivo* tracking agent enabled a direct and reliable correlation between *in vivo* whole body imaging by ^19^F-MRI and *ex vivo* localization in tissues by Raman microscopy.

## Results and Discussion

### Imaging Properties of Fluorinated Probe Nanoformulations

**Figure 2:**
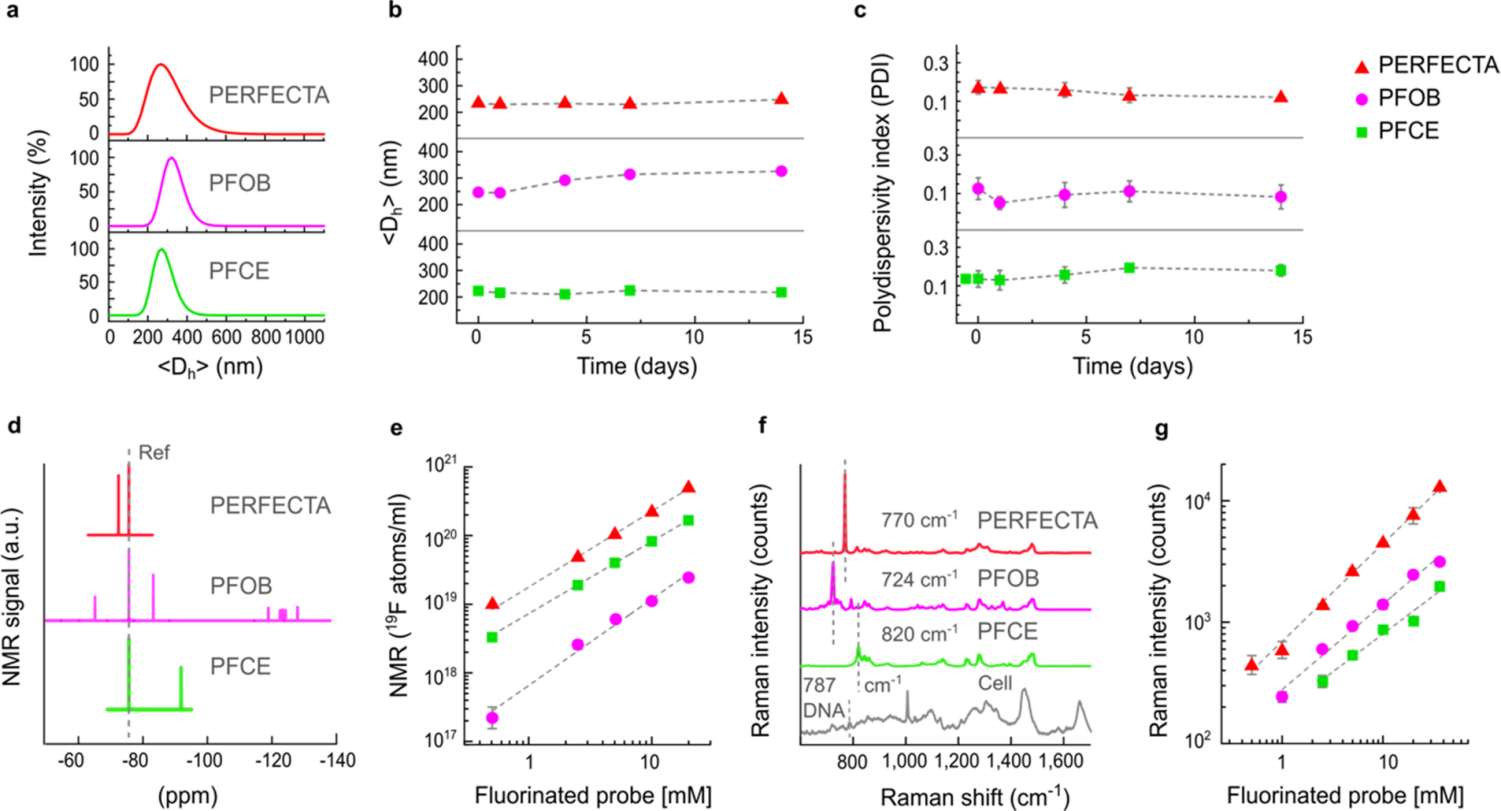
Raman and NMR/MRI characteristics of ^19^F-probes. (a–c) Nanoformulation features and stability in water at room temperature (25 °C). (a) Shelf life of each set of nanoformulations was explored monitoring their DLS behavior over time by plotting the Z-averaged hydrodynamic diameters, *D*_h_ (b), and PDIs (c) versus time, up to 2 weeks. (d) ^19^F-NMR spectra of nanoformulations of PERFECTA or PFOB or PFCE acquired together with a reference (dashed line). (e) Intensity dependence of the ^19^F-NMR signal on fluorinated probe concentration calculated as number of ^19^F/mL. (f) Raman spectra of each fluorinated probe nanoformulation and typical Raman spectrum of mammal cells (multiplied two times for clarity). (g) Intensity dependence of the Raman signals at 770, 724, and 820 cm^–1^ for PERFECTA, PFOB, and PFCE, respectively, versus fluorinated probe concentration.

The bioimaging features of PERFECTA in both modalities are here shown in comparison to two other commonly used fluorinated probes, i.e., PFCE and PFOB (Figure 1). Biocompatible nanformulations of these three fluorinated probes were prepared in aqueous solution and stabilized by the nonionic surfactant PluronicF68, showing similar size distributions, surface charges, and colloidal stabilities (Figure 2a–c and Table S1). These measurements were performed in water at room temperature (25 °C). Further studies were also assessed in biological fluids at 37 °C, and results confirmed the NPs stability at least up to 72 h in all investigated media (Figure S1a–f). All these nanoformulations, which proved successful as labeling agents for cell tracking and *in vivo* imaging by ^19^F-MRI,^27,28^ thanks to their distinctive ^19^F-NMR signals, are also characterized by important Raman features related to their molecular structures, although with substantial differences (Figure 2d–g).

Regarding ^19^F-NMR properties, PERFECTA gave a single sharp and intense signal in the ^19^F-NMR spectrum at −73.4 ppm, and its superior intensity is clearly related to the highest fluorine payload (36 equiv of ^19^F atoms per molecule) (Figure 2d,e). Similarly, PFCE gave an intense and characteristic single peak due to its 20 equiv of ^19^F atoms, while PFOB exhibited a ^19^F-NMR spectrum with multiple peaks that reduced overall response (Figure 2d,e). Concerning Raman properties (Figure 2f), PERFECTA exhibited a sharp and specific Raman peak at 770 cm^–1^, with the highest intensity among tested fluorinated probes, proportional to its molar concentration (Figure 2g). The molecular symmetry of PERFECTA accounts for its strong and narrow Raman peak intensity that is assigned to collective stretching and bending vibrations, symmetrically replicated over the four arms of the molecule, as demonstrated by density functional theory (DFT) simulations (Figures S3, S4). Similarly, the Raman marker of PFOB (724 cm^–1^) is assigned to collective symmetric stretching and bending vibrations, exhibiting a good intensity, whereas the marker of PFCE (820 cm^–1^) is assigned to collective symmetric bending (Figures S3–S5), which explains its reduced intensity compared to those obtained with the other fluorinated probes. Since the molecular structures of both PFOB and PFCE are conformationally more flexible than PERFECTA, their Raman bands not only are less intense but also have a broad line width. Importantly, despite that the most intense Raman peaks of all tested fluorinated probes fall in a relatively silent biological region, between 500 and 800 cm^–1^, it also includes the typical DNA vibrational modes (around 790 cm^–1^). However, DNA detection is not compromised in biological samples, as we show in detail in Figure 1 and Figure 2f. Moreover, an estimated limit of detection in cell culture medium for PERFECTA has been estimated as 0.3 mM (Figure S2).

### Bimodal Imaging of Cells Labeled with Fluorinated Probes

**Figure 3:**
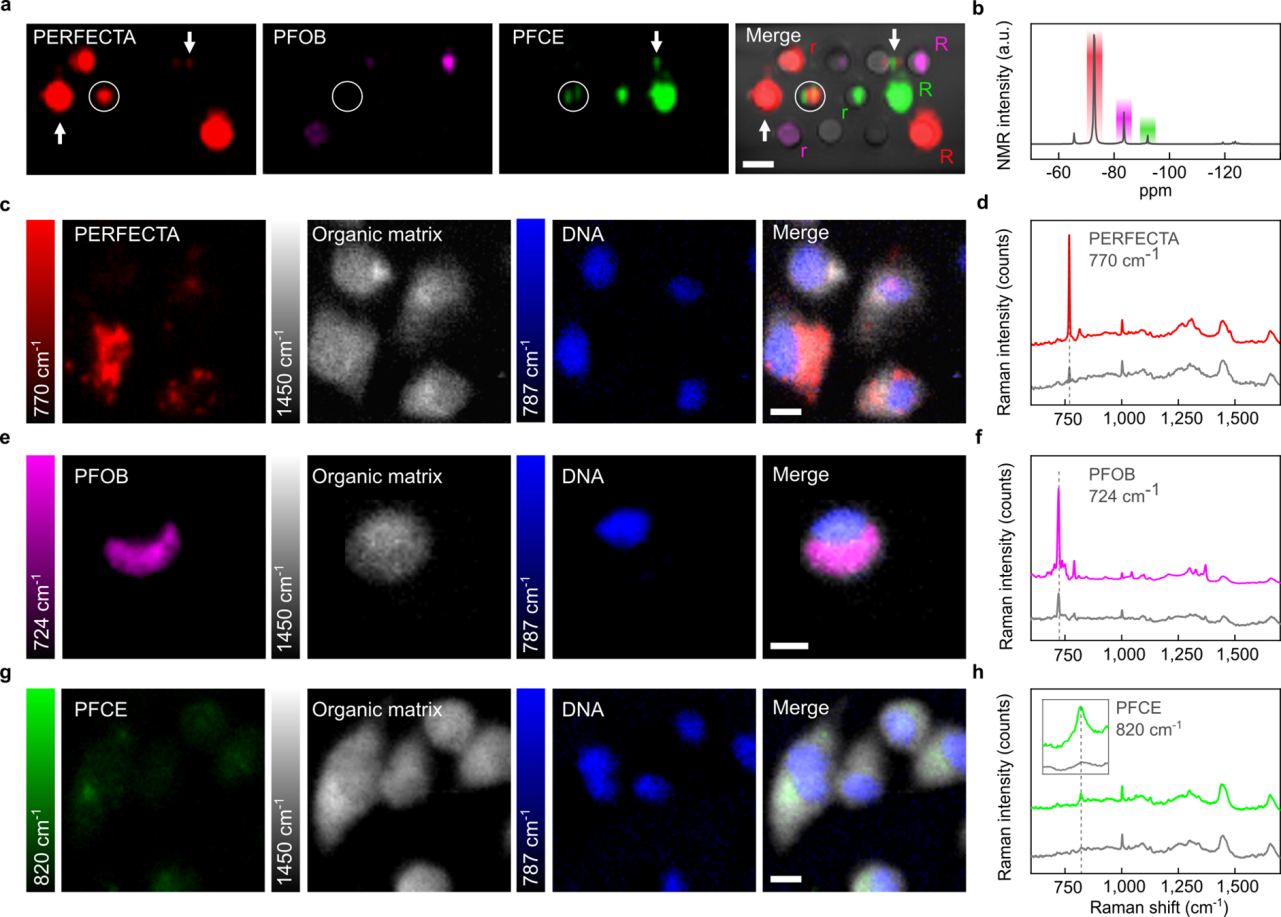
Raman and ^19^F-MRI multicolor imaging of labeled cells. (a) ^19^F-MRI of a phantom containing a mix of murine microglial cells (white circle) incubated separately with each fluorinated probe nanoformulation and acquired at the main ^19^F-chemical shift frequency of each probe (35 min per scan with a voxel size of 0.7 × 0.9 × 3 mm^3^) together with samples of each nanoformulation (R: 5 × 10^19^ atoms and r: 2.5 × 10^19^ atoms for each respective probe). ^19^F-MR images were merged with the ^1^H-MR image (gray) for sample localization (merge). Unexpected signals (arrows) due to chemical shifts of PFOB are visible in images acquired at frequencies of PERFECTA and PFCE. Scale bar = 5 mm. (b) ^19^F-MR spectrum of the entire set of samples (mixed cells and relative references of each fluorinated probes) acquired during MRI experiments with the selected frequency band reported for each fluorinated probe. (c, e, g) Raman imaging of *in vitro* labeled murine microglial cells with nanoformulations of PERFECTA, PFOB, and PFCE, respectively, after selecting Raman bands of each fluorinated probe, organic matrix (1450 cm^–1^), and DNA (787 cm^–1^) (scale bar, 10 μm). (d, f, h) Raman spectra of the cellular regions presenting signals of PERFECTA, PFOB, and PFCE, respectively, and of the cytoplasmic regions (organic matrix, gray) from imaged cells reported in panels c, e, and g. Raman maps were collected in raster scan modality using a step size of 0.7 μm and by two acquisitions of 1.2 s for each step. Raman spectra were obtained by averaging pixels mainly associated with each signal. (a.u.: arbitrary unit).

**Figure 4:**
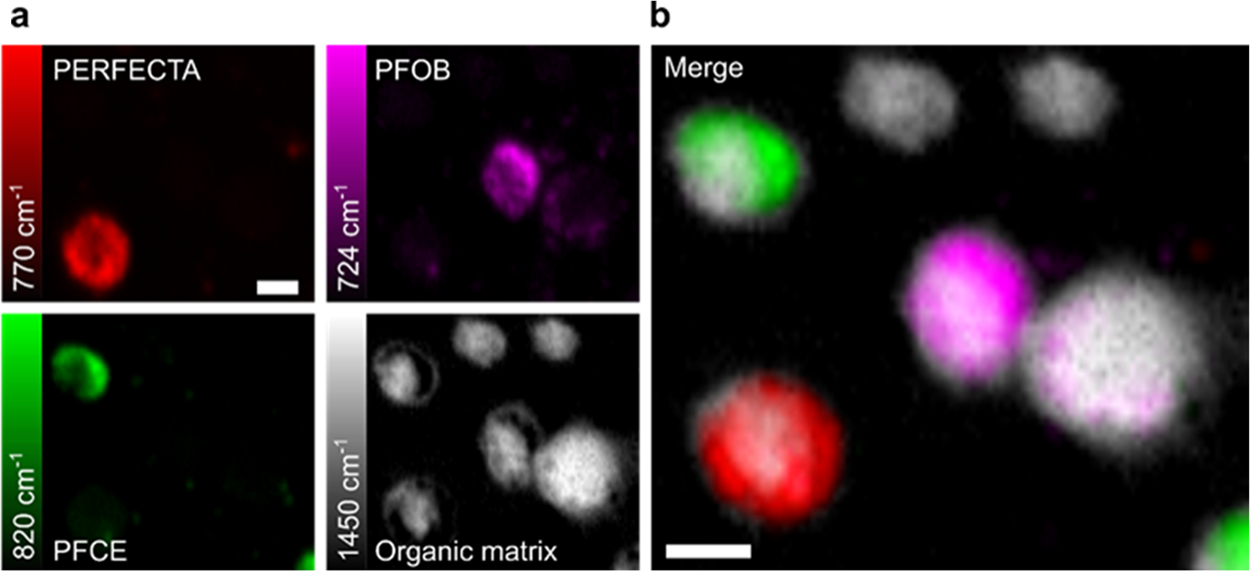
*In vitro* tricolored Raman imaging of ^19^F-labeled cells. Labeled cells were separately incubated with analogue formulations of PERFECTA, PFOB, and PFCE at the same concentration of fluorine (1.9 × 10^14 19^F atoms/cell) and then mixed. (a) Raman imaging was acquired after selecting Raman bands of each fluorinated molecule and organic matrix (1450 cm^–1^). (b) A tricolored image was obtained by merging the images acquired at each band (scale bar, 10 μm). Raman maps were collected in raster scan modality using a step size of 0.7 μm and by two acquisitions of 1.2 s for each step.

Starting from this first evidence originated by the characterization of the tested fluorinated probes, *in vitro* experiments in immune effector cells of the brain were performed to screen and evaluate the three fluorinated probes. Thanks to their phagocytic capacity, murine microglial cells were easily labeled with each fluorinated probe separately, at a fixed fluorine dose, and then imaged. As shown on ^19^F-MR images acquired at the NMR frequency of each fluorinated probe (Figure 3a,b), the pool of mixed labeled cells was clearly detected on images of PERFECTA and PFCE, together with appropriate references. Although a tricolored MRI was obtained by combination of each acquired image, cells labeled with PFOB were barely detectable and the multiplets of PFOB induced unexpected fluorine signals on images of PERFECTA and PFCE in areas that do not contain the relative fluorinated probes (Figure 3a). Such artifacts, due to off-resonance chemical shifts, are commonly observed with standard MRI acquisitions and could be eliminated by using dedicated MRI methods that have been recently proposed to improve PFOB detection.^29,30^ The same labeled cells were imaged by Raman microscopy selecting the specific Raman peaks of PERFECTA, PFOB, and PFCE (770, 724, and 820 cm^–1^, respectively) (Figure 3c–h). All three fluorinated probes were clearly detected at the intracellular level in the cytoplasm, as also confirmed by confocal microscopy by using fluorinated probes nanoformulated together with fluorescent dyes (Figure S6a–c), but Raman microscopy allowed the direct intracellular visualization of the same fluorinated molecule detected by MRI. Moreover, as suggested by spectral analysis, we confirm that the Raman signal from each fluorinated probe does not prevent the detection of label-free intrinsic chemical contrast of key cellular components such as the nuclear DNA. Remarkably, signals of both PERFECTA and PFOB were strong and clearly distributed in the cytoplasm, whereas PFCE was more difficult to detect due to its broader and less intense Raman signal. Of note, similarly to the use of fluorophores in fluorescence microscopy, tricolored Raman was obtained on a mixture of cells treated separately with each fluorinated probe (Figure 4), suggesting the potential use for multiplexing and multitargeting imaging.^31^ As emerged from these data, if, on one hand, all the fluorinated probes were clearly visible intracellularly, PERFECTA outperformed in both modalities in terms of signal intensity. In fact, PFOB showed a relatively good Raman signal, but it lacked sufficient MRI sensitivity and specificity, while PFCE was a good MRI probe, but it gave a weak Raman signal, which was comparable to the cell background. For these reasons, PERFECTA was then selected as appropriate bright bimodal probe for *in vivo* MRI, followed by *ex vivo* Raman microscopic identification in tissues.

### From *in Vivo* Whole Body Imaging to Tissue Microscopy

Besides tracking *in vitro* labeled cells by MRI, fluorinated probes have been widely used for imaging inflammatory activity by *in vivo* labeling following systemic administration. Indeed, nanoformulations of fluorinated probes are avidly taken up by phagocytes as leukocytes^27,32−35^ that are highly present in injured tissues or organs with an immune reaction.

**Figure 5:**
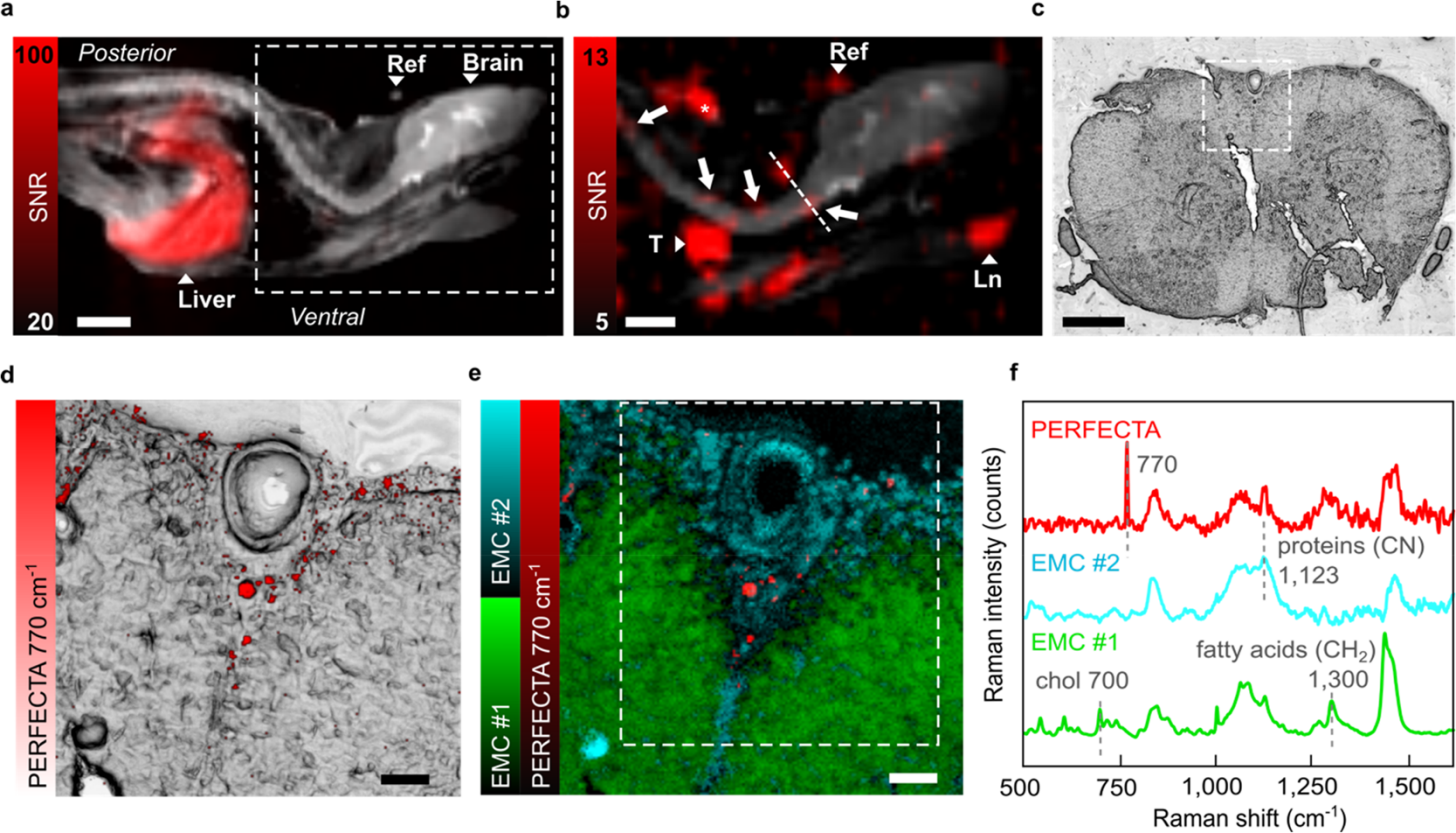
From *in vivo* to *ex vivo* bimodal imaging with the bioorthogonal ^19^F-probe. (a) *In vivo*^19^F-MRI at the specific resonance frequency of PERFECTA (red color scale) was acquired (36 min with a voxel size of 0.72 × 0.87 × 1.5 mm^3^) over the mouse body and merged with the anatomical image (^1^H-MRI, gray color; ref: external reference of PERFECTA). Scale bar, 5 mm. A uniform and intense signal of PERFECTA was found in the entire liver (L), lymphoid organs (b: T, thymus, and Ln, cervical lymph nodes) and in the area of immunization for EAE induction (*). (b) Several red spots were observed along the spinal cord (white arrows) where Raman imaging was successively performed. Scale bar, 5 mm. (c) Bright field (BF) image of the unstained fresh-frozen tissue section collected from mouse spinal cord (cervical region) and studied by Raman imaging (white dashed square). Scale bar, 0.5 mm. (d) BF image of the selected tissue regions merged with the Raman map of the PERFECTA band. (e) Merging of Raman maps related to empty modeling component (EMC) #1 and #2 and to PERFECTA. (f) Typical (averaged) Raman spectra obtained by *ex vivo* Raman imaging of tissue slices. EMC #1 mostly represents white matter (green) (chol: cholesterol); EMC #2 mostly represents epithelium and immune cell infiltration (cyan); PERFECTA spectrum is the average of image pixels reporting a signal at 770 cm^–1^. Raman maps on tissue slices were collected in raster scan modality using a step size of 2.5 μm and a single 2 s acquisition for each step. For d and e, scale bar = 50 μm.

For this reason, the bimodal properties of the PERFECTA nanoformulation were tested *in vivo* in the well-established autoimmune encephalitis (EAE) mouse model that develops multifocal neuroinflammation along the spinal cord,^36^ similarly to patients affected by multiple sclerosis. From *in vivo*^19^F-MRI of mice treated with the PERFECTA nanoformulation, most of the ^19^F signal from PERFECTA was found in the liver (Figure 5a), but also along the spinal cord as small hot spots (Figure 5b), where leukocyte infiltrates were expected to be found in this model.^36^ Moreover, a PERFECTA signal was also detected in lymphoid organs as thymus and cervical lymph nodes (Figure 5b), which are particularly active during EAE. Furthermore, using an external reference, the PERFECTA signal measured in the spinal cord corresponded to a concentration between 0.2 and 3 mM according to tested mice (*n* = 3). Following *in vivo* MRI, sections of spinal cord were collected and directly analyzed by Raman microscopy without further labeling or staining (Figure 5c–f). This analysis allowed the direct localization of PERFECTA Raman signatures in the tissue, thus validating *in vivo* MRI observations, but also the assessment in label-free modality of the biomolecular composition of the tissue where the fluorinated probes were localized. For instance, PERFECTA signals (Figure 5d,e) were detected proximal to a vessel in the ventral part of the spinal cord, with Raman features typically associated with a relatively high cellularity, specifically with a reduced lipidic content and high protein-related signals (Figure 5e). Indeed, PERFECTA was not found in regions rich in fatty acids, cholesterol, and other lipids, mostly associated with myelin and healthy white matter (Figure 5e). These observations are in agreement with the pathological characteristics, in which immune cell infiltrations are associated with demyelination of white matter tracts.^36^ As expected, the signals from the fluorescent dye Dil, inserted on purpose in PERFECTA nanoformulation, colocalize with the leukocyte marker (CD45), as shown by immunofluorescence microscopy in adjacent tissue slices (Figure S7). Interestingly, Raman images were also obtained in an EAE mouse with a reduced concentration of PERFECTA (estimated by ^19^F-MRI about 0.2 mM) in the spinal cord at the limit of detection for both imaging modalities (Figure S8).

## Conclusions

We showed that the superfluorinated ^19^F-MRI probe PERFECTA has also a strong, specific, and stable over time Raman signal thanks to its characteristic C–F bonds, thus allowing a direct and reliable correlation between the two imaging modalities, which is crucial for the validation and development of imaging probes. This signal falls in a relatively silent biological region, making it biorthogonal to the biological matter. Intracellular uptake of PERFECTA is demonstrated without use of additional fluorescent dyes. Thus, this enables the use of PERFECTA as a bright bimodal probe for both macroscopic *in vivo* imaging across the entire body and organs by ^19^F-MRI and microscopic imaging at histological and cellular levels by Raman microscopy. Of note PERFECTA shows unparalleled performances in both imaging modalities when compared to other fluorinated probes, such as PFCE and PFOB. On the other hand, the three probes show separated signals, and simultaneous intracellular detection of the three fluorinated probes has been achieved, indicating the possibility to use these probes for multiplexing imaging.

Commonly used bimodal probes have to overcome the complex synthesis needed to combine two different molecular moieties in the same structure (i.e., MRI and fluorescent dyes) and their potential differences in pharmacokinetics. The use of a unique molecule as bimodal probe not only permits reliable correlations but also avoids misinterpretations and facilitates the translation for clinical purposes. For instance, a single bimodal probe could be used to identify by MRI the tumor for surgical extraction or biopsy and to further characterize the tissue by intraoperative or *ex vivo* Raman imaging.^37,38^ Importantly, the use of Raman microscopy allows localization of the tracking agent at the tissue level and also permits obtaining unique additional information on the biomolecular composition of the biological environment, distinguishing healthy tissues from diseased regions. PERFECTA shows a peculiar advantage over other Raman probes described in the scientific literature. SERS tags are, in fact, highly bright and can be detected at a single-probe level.^10^ However, they usually have a complex spectrum characterized by multiple peaks and fluorescence background hiding signals from the surrounding tissue. Alkyne and deuterium tags have instead simpler spectra and can be easily incorporated into the sample, but their cross-sections are very small, and they are challenging to detect by Raman microscopy. In this regard, PERFECTA, with its 36 carbon–fluorine bonds, displays a single Raman peak at 770 cm^–1^ with a quite high cross-section. Furthermore, besides its unique spectroscopic and magnetic properties, PERFECTA has also an intrinsic chemical versatility for further functionalization, differently from other fluorinated probes. Its molecular design allows substitution of one of the four symmetrical arms with an alkyl chain bearing different functional groups such as thiol,^39^ carboxylic acid, alcohol, azide, and amine, still maintaining 27 magnetically equivalent ^19^F in the molecule. This versatility paves the way for the development of a new set of dual-imaging functional tags, based on a library of PERFECTA derivatives, for detecting biological targets by ^19^F-MRI and Raman microscopy. Finally, given the strong stretching vibrations of the C–F bond in the 1000–1400 cm^–1^ region, these tags may also be foreseen as active IR markers suitable for the emerging mid-infrared (MIR) microscopy^40^ technology, which will be studied in the near future.

## Supporting Information Available

Additional experimental and computational details, analytical graphs, supplementary figures, and references, including Table S1 and Figures S1–S8 (PDF)
