# Supplementary material for: A Bioorthogonal Probe for Multiscale Imaging by 19F-MRI and Raman Microscopy: From Whole Body to Single Cells
Source: J Am Chem Soc. 2021 Jul 28;143(31):12253–60. doi: 10.1021/jacs.1c05250 (PMC8397317; doi:10.1021/jacs.1c05250)
Supplement: Supplementary file 1 — ja1c05250_si_001.pdf [file ja1c05250_si_001.pdf]

SUPPORTING INFORMATION

A Bioorthogonal Probe for Multiscale Imaging by ^{19}F-MRI and Raman Microscopy: From Whole Body to Single Cells

Cristina Chirizzi[1†], Carlo Morasso[2†], Alessandro Aldo Caldarone[2], Matteo Tommasini[3], Fabio Corsi[2,4], Linda Chaabane[5*], Renzo Vanna[6*], Francesca Baldelli Bombelli[1*] and Pierangelo Metrangolo[1]

1. Laboratory of Supramolecular and Bio-Nanomaterials (SupraBioNanoLab), Department of Chemistry, Materials, and Chemical Engineering "Giulio Natta", Politecnico di Milano, Via Luigi Mancinelli 7, 20131 Milan, Italy.
2. Istituti Clinici Scientifici Maugeri IRCCS, via S. Maugeri 4, 27100 Pavia, Italy.
3. Department of Chemistry, Materials, and Chemical Engineering "Giulio Natta", Politecnico di Milano, Via Luigi Mancinelli 7, 20131 Milan, Italy.
4. Department of Biomedical and Clinical Sciences "Luigi Sacco", Università di Milano, via G. B. Grassi 74; 20157 Milan, Italy
5. Experimental Neurology (INSPE) and Experimental Imaging Center (CIS), Neuroscience Division, IRCCS
6. CNR-Institute for Photonics and Nanotechnologies (IFN-CNR), Department of Physics, Politecnico di Milano, Piazza Leonardo Da Vinci 32, 20133 Milan, Italy

*Linda Chaabane: chaabane.linda@hsr.it

*Renzo Vanna: renzo.vanna@ifn.cnr.it

*Francesca Baldelli Bombelli: francesca.baldelli@polimi.it

Contents

1. Additional experimental details, materials and methods 3

1.1. Preparation of nanoformulated ^{19}F-probes 3

1.2. Nanoparticles characterization 3

1.3. Density Functional Theory (DFT) simulations of Raman spectra 3

1.4. NMR Experiments 3

1.5. MRI Protocol 4

1.6. Raman spectroscopy and Raman imaging 4

1.7. *In-Vitro* Cell Labelling 5

1.8. Animal Experiments 5

1.9. Tissue preparation 6

1.10. Immunohistochemical microscopy 6

2. Chemical features of Fluorinated probes 7

3. Colloidal stability of nanoparticles in different media 8

4. Determination of PERFECTA limit of Detection (LOD) by RAMAN 9

5. Simulation of Raman Spectra by DFT calculations 10

5.1. Equilibrium structures of the fluorinated probes 10

5.2. DFT-simulated RAMAN spectra 11

5.3. DFT optimized molecular structures 17

6. Fluorescence imaging studies 18

6.1. Confocal microscopy on labeled cells 18

6.2. Confocal microscopy on tissue sections 18

7. From *in-vivo* MRI to tissue RAMAN microscopy 19

8. Supplementary references 20

1. Additional experimental details, materials and methods

1.1. Preparation of nanoformulated ^{19}F-probes. PERFECTA was synthesized as previously described[1], while perfluoro-15-crown-5-ether (PFCE) and perfluorooctyl bromide (PFOB) were supplied by Exfluor Research Corporation, Round Rock, TX, USA and Apollo Scientific Ltd, UK, respectively. We followed an optimized protocol previously reported[2], where the nanoformulations containing the three distinct fluorinated probes were obtained forming an emulsion with a non-ionic surfactant (Pluronic F68, Serva, Heidelberg, FRG). Briefly, the aqueous solution of the surfactant (Pluronic F68) was prepared adding the copolymer, in form of powder, to sterile water and keeping the mixture under magnetic stirring at 25°C. PFCE (16.6% w/v), PERFECTA (7.5% w/v) or PFOB (20% v/v) were emulsified with the surfactant solution (9.3 % w/v) through three consecutive on-off cycles of tip-sonication with an ultrasonic pulse duration, t_p= 90 s. For PERFECTA, the water phase was also heated to 80°C and quickly mixed with the perfluorocarbon previously kept at the same temperature. Several cycles of heating and sonication were carried out to reach homogeneous formulations. In some cases, the nanoformulations were also fluorescently labelled by addition, at the last sonication cycle, of different dyes at a fixed concentration (200 μM) for each set of nanoformulations (DiOC18(3), ThermoFisher SCIENTIFIC for PFCE; DiIC18(3) Sigma Aldrich for PERFECTA and PFOB), for performing confocal microscopy analysis. The free dye content was removed by centrifugation (10000 × *g* for 20 min).

1.2. Nanoparticle characterization. The size distribution of the nanoformulations was evaluated by multi-angle Dynamic Light Scattering (DLS) using an ALV apparatus equipped with ALV- 5000/EPP Correlator, special optical fiber detector and ALV/CGS-3 Compact goniometer. The light source is He–Ne laser (λ=633 nm), 22 mW output power. Zeta-averaged hydrodynamic diameters (D_H) were obtained by a cumulant fitting of the auto-correlation functions. For these analyses, the stock preparation was diluted 1:50 (v/v) in water and measured at room temperature (25°C).The shelf life of the formulations was assessed by daily measures of size by DLS on samples stored at 4 °C. The mean diameters and the polydispersity index (PDI) of all nanoformulations were stable up to 2 weeks (Fig. 2b-c). Colloidal stability of the three nanoformulations in biological fluids was assessed diluting each formulation 1:6 (v/v) in water (ctrl), DMEM and solutions of FBS at 10% in DMEM, and incubating the samples at 37 °C. The surface charge of the nanoparticles was measured using a Litesizer 500 Anton Paar Instruments, Graz, Austria. All measurements were performed in PBS 1X using a sample dilution 1:50 (v/v) (Table S1).

1.3. Density Functional Theory (DFT) simulations of Raman spectra. The Gaussian09 suite of quantum chemistry codes was used to compute the off-resonance Raman spectra of PERFECTA, PFCE and PFOB by the DFT method B3LYP/6-311G(d,p). The input conformation of PERFECTA was obtained from X-ray diffraction data[1]. The assessment of the lowest energy conformations of PFCE and PFOB was carried out with the help of the Avogadro software (version 1.2.0) and the MMFF94 molecular mechanics force field. The conformational search tools of Avogadro allowed to prepare the input structure for the DFT frequency calculations of PFCE and PFOB. The three structures reported in Fig. S1 are stable minima, as proved by the absence of negative eigenvalues of the computed Hessian matrix.

1.4. NMR Experiments. To evaluate the ^{19}F-content in the nanoformulations, a capillary containing 100 μl of 0.35M trifluoroacetic acid (TFA) in D_2O as internal standard solution was added to 400 μl of diluted nanoformulation sample. The preparation of the sample was followed by acquisition of

NMR spectra on a Bruker Avance III HD 400 spectrometer (Bruker BioSpin, Rheinstetten, Germany) using a Bruker Automatic Sample Changer (SampleXpress). ^{19}F NMR spectra were recorded in the range of −8 to −138 ppm (CF_3COOH as reference, ppm) relative to TFA (-75 ppm). The NMR probe was maintained at 300 K during the whole experiment. A delay time between repetitions of 14.5 s was adopted to ensure full relaxation and 256 scans were collected, reaching a total acquisition time equal to 14 minutes. All ^{19}F NMR spectra were automatically phased and baseline corrected for accurate quantitative analysis through MestReNova software. Each set of nanoformulation was characterized via ^{19}F-NMR in order to quantify the fluorine content. In detail, ^{19}F-NMR spectra were done on diluted sample in mQW using TFA reference previously described. From the ratio of integrals from each peaks, the fluorine content was calculated (Fig. 2d). We found an average number of fluorine atoms equal to 1.85, 0.8 and 3.7 x 10^{21}/ml, which corresponds to a 153.6, 36.7 and 361.4 mM concentration of fluorinated tracer in each preparation for PFCE, PERFECTA and PFOB, respectively.

1.5. MRI Protocol. All experiments were performed on a 7T scanner (Biospec; Bruker-Biospin) using a dual-transmit receive $^{19}F/^{1}H$ volume coil equipped with a horizontal bore magnet (ultra-shielded). ^{19}F-MR images were acquired at the specific resonance frequency of each ^{19}F-probe determined from ^{19}F MR spectroscopy (Table S1). For PFOB, images were acquired at the frequency with the highest peak intensity (ie. -83.2 ppm).

For both *in vivo* and *in vitro* acquisitions, a 3D turbo-spin echo sequence was used with the same field of view (45×30×24 mm) for ^{1}H-MRI (TR/TE=250/15 msec, matrix=128×64×8, 6 averages) and for ^{19}F-MRI (TR/TE=1500/40 msec, 64×32×8, 32 averages). Both ^{1}H and ^{19}F MRI data were merged using Image-J software (https://imagej.nih.gov/ij/).For *in vivo* MRI, a mixture of IsoVet (1-2%; Zootecnica) with oxygen was used to anaesthetize animals and breath rate was continuously monitored to adjust the level of anaesthesia. Body temperature of mice was maintained through warm water circulating inside the bed.

1.6. Raman spectroscopy and Raman imaging. All Raman data were collected using a commercial confocal Raman microscope (InVia Reflex, Renishaw plc, Wotton-under-Edge, UK) coupled with a 785 nm excitation laser diode source (90 mW) then filtered to reach around 20mW on the sample. The back-scattered Raman light was collected by a N-Plan 100x (NA 0.75) Leica objective for the study of nanoformulation and tissue samples, and by a LUMPlanFl/IR 60x/W (NA 0.90) Olympus objective for the study of cells. The Raman photons were then diffracted by a 1200 l/mm grating, cantered around 1100 cm^{-1} thus giving a spectral range between 650 and 1650 cm-1. The photons were read by a CCD (1024x256) cooled at -70°C. The instrument was automatically aligned and calibrated before each experiment using the silica band at 520 cm^{-1} and periodically fully calibrated by multiple standards (polystyrene, paracetamol, silica). Data analysis, statistical data analysis and plotting were performed using WiRe software (Renishaw plc, Wotton-under-Edge, UK), MATLAB (MathWorks, Natick, MA, USA) or OriginPro2019 (Originlab Corporation, Wellesley Hills, MA, USA).

RAMAN properties of each fluorinated probes were verified performing specific spectra of nanoformulations by drying a small drop of dispersion (typically 5 µL) on CaF_2 discs (Crystran, UK). Spectra were collected for 30 seconds. Five different measures were acquired on each sample. Spectra were vector normalized and the average of the different measures was considered as the final spectrum for each nanoformulation.

For Raman imaging on cells, Raman maps (with average size set to 60x60 μm^2) were collected in raster scan modality using a step-size of 0.7 µm and by two acquisitions of 1.2 s for each step, positioning the laser spot focus at around 4 µm above the CaF_2 disk. Each Raman map was pre-processed for cosmic rays removal (nearest neighbour and width of features algorithms) and any

other pre-processing procedure was carried out. False-colour Raman images were achieved by merging different channels related to cell cytoplasm, cell nucleus and PFC/NP, using ImageJ2/Fiji. The band around 1450 cm^{-1} (organic matrix) was used to detect the cytoplasm, the band around 787 cm^{-1} (DNA) was used to detect the nucleus, and the most intense Raman peaks related to the three fluorinated probes (770 cm^{-1} for PERFECTA, 820 cm^{-1} for PFCE, and 724 cm^{-1} for PFOB, respectively) were used to localize fluorinated probes.

Finally, Raman maps on tissue slices (with average size set to 400x400 μm^2) were collected in raster scan modality using a step size of 2.5 µm and a single 2 s acquisition for each step, positioning the laser spot focus around 2 µm below the tissue surface. Each Raman map was pre-processed and analysed as described for Raman imaging of cells and adding baseline subtraction (12 degrees polynomial fitting) to remove endogenous tissue autofluorescence. To produce Raman images on tissue an empty modelling (EM) k-means based clustering algorithm (Renishaw plc, Wotton-under-Edge, UK) was applied for the detection of the first two EM-components (EMC) to automatically localize pixels associated to main tissue features. The average spectra of pixels associated to the these two EMCs were extracted. The localization of PERFECTA was performed by selecting pixels with signal at 770 cm^{-1}.

1.7. *In-Vitro* Cell Labelling. The immortalized murine microglial cell-line, BV-2 (kindly provided by Dr. R. Furlan, IRCCS Ospedale San Raffaele), was used as a model of phagocytic cells suitable for cell labelling assays. BV-2 cells were maintained in Dulbecco's modified Eagle's medium (DMEM; Lonza), containing 10% fetal bovine serum, 100 mg/ml streptomycin, 100 U/ml penicillin and 2 mM glutamine (Gibco-Invitrogen). Microglial cells were seeded on 6-multiwell plates (1×10^6 cells/well) and incubated with the nanoformulations at a concentration of 1.2×10^{20} ^{19}F/ml for 4 h at 37 °C in 5% CO_2. Cells were then washed with phosphate buffered saline (PBS) to remove the excess of not phagocytosed nanoparticles. Cell viability was assessed by standard trypan blue exclusion method. As a control, cells without ^{19}F probes followed the same procedures.

For confocal microscopy, cells were incubated on poly-L-lysine-coated glass coverslips and fixed in 4% PFA. Fixed cells were labelled with 4′-6-diamidino-2-phenylindole (DAPI) for nucleus staining and imaged at the Leica SP5 confocal microscope (Leica Microsystems, Milan, Italy).

For MRI, 3 x 10^5 of labelled cells were fixed in 4% paraformaldehyde and washed. Pellets of cells were produced by centrifugation and placed in tubes prefilled with 2% agar.

For Raman imaging, 4 x 10^5 microglial cells were seeded on CaF_2 discs pre-treated with poly-lysine 0.01% (40 min ant 37°C). After ^{19}F labelling, cells were washed twice with PBS, fixed by 2% PFA in PBS and stored at 4 °C.

1.8. Animal Experiments. Six- to eight-week-old C57Bl/6 female mice were purchased from Charles River Laboratories (Calco, Italy). All animal experiments were performed in accordance with the national guidelines on animal care approved by the Institutional Animal Care and Use Committee (IACUC) at San Raffaele Scientific Institute (Milan, Italy).

Chronic autoimmune encephalomyelitis (EAE) was induced in female C57BL/6 mice, by subcutaneous with 300 µl of 200 µg per mouse of $MOG_{35–55}$ in Freund's Adjuvant Incomplete liquid, IFA, (Sigma) supplemented with 8 mg ml^{-1} *Mycobacterium tuberculosis* (strain H37Ra; Difco, Lawrence, KS, USA). Pertussis toxin (500 ng, List Biological Laboratories, Campbell, CA, USA) was intravenously injected on the day of the immunization and again 2 days later.

In-vivo ^{19}F-MRI was performed after 2 weeks from the intraperitoneally administration of PERFECTA-nanoformulation (6.4×10^{20} ^{19}F/mouse), given at the acute phase of clinical symptoms

(20 days post immunization, clinical score of 2.5-3.5). As control, healthy mice followed the same treatment.

Clinical assessment of EAE was performed according to the following scoring criteria: 0 = healthy, 1 = limp tail, 2 = ataxia and/or paresis of hind limbs, 3 = paralysis of hind limbs and/or paresis of forelimbs, 4 = tetraparalysis, and 5 = moribund or death.

At the end of the experiment, animals were euthanized and perfused with the aim to collect different organs for immunohistological and RAMAN spectroscopy analysis.

1.9. Tissue preparation. For immunofluorescence and Raman Imaging of the spinal cord, mice were transcardially perfused with saline buffer plus EDTA 0.5 M following terminal anaesthesia. Perfusion was continued with cold 4% paraformaldehyde (PFA).

The spinal-cord was carefully extracted from the bones and post-fixed overnight in 4% PFA at 4 °C. Tissues were then cryoprotected with sucrose, sectioned in 12 µm thick slices on a cryostat and processed for fluorescent microscopy experiments. For Raman imaging, contiguous 10µm tissue slices were microtomed from OCT fixed frozen tissue blocks and mounted on mirrored stainless-steel slides (Renishaw plc, Wotton-under-Edge, UK).

1.10. Immunohistochemical microscopy. Tissue sections were stained using an antibody against CD45 (Biolegend, USD) to identify leucocytes. In detail, samples were washed with PBS and incubated in blocking solution containing 10% serum of secondary Ab species in PBS at room temperature. Each section was incubated with antibodies against CD45 overnight at 4 °C.

The following day CNS slices were rinsed in PBS and fluorescent secondary antibodies (conjugated with Alexa Fluor 647), diluted in blocking mix. Slides were then washed in PBS and stained with DAPI for nuclei counterstaining (Roche Diagnostics Spa, Monza, Italy). The Leica SP5 and SP8 (Leica Microsystems, Milan, Italy) were used for image acquisitions. Images were analysed with ImageJ software (NIH).

2. Chemical features of the fluorinated probes

	PERFECTA	Perfluoro-15-crown-5-ether (PFCE)	Perfluorooctyl bromide (PFOB)
**Chemical formula**	$C_{21} H_8 F_{36} O_4$	$C_{10} F_{20} O_5$	$C_8 F_{17} Br$
**Molecular weight (g/mol)**	1008.2	580.1	497.9
**Nanoformulation diameter (nm)** **[PDI]**	234 ± 23 [0.15]	218 ± 22 [0.16]	284 ± 28 [0.09]
**Mean zeta potential (mV) ***	- 0.5 ± 0.2	- 0.5 ± 0.2	- 0.6 ± 0.2
**NMR chemical shifts (ppm)**	-73.4	-92.7	-65.1, -**83.2**, -118.9, -112.5, -123.2, -124.2, -127.9
**RAMAN (cm^{-1})**	770	724	820

Table S1. Summary on properties of the nanoprobes used for *in vitro* experiments

***** These measurements were performed in PBS 1X.

3. Colloidal stability of nanoparticles in different media

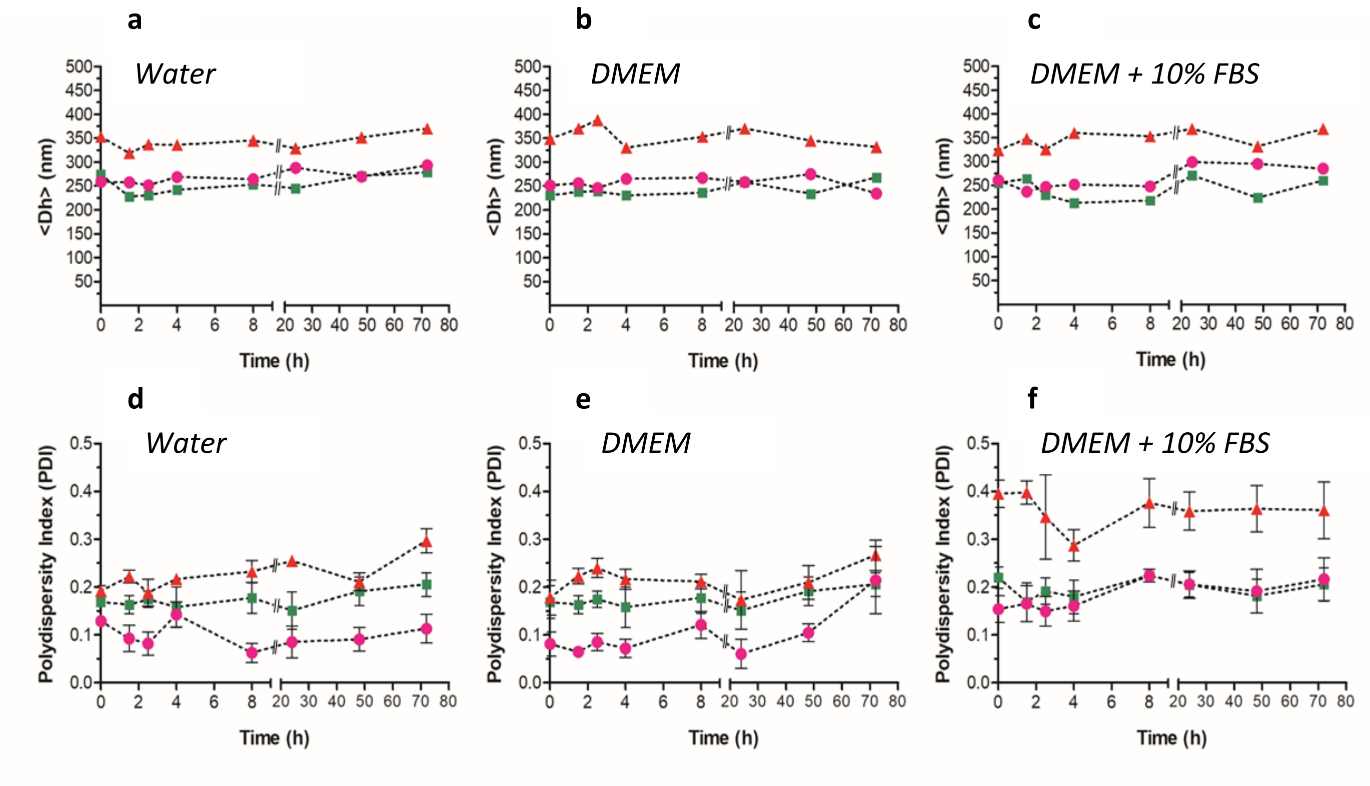

Figure S1. Colloidal nanoparticles stability in different media. a-c: Plot of Zeta-averaged hydrodynamic diameter versus time in water, DMEM and DMEM supplemented with 10% FBS (fetal bovine serum), respectively. **d-f**: PDIs versus time. (red triangle: PERFECTA, magenta circle: PFOB and green square: PFCE). Stability in biological fluids was assessed diluting each formulation 1:6 (v/v) in water (ctrl), DMEM and solutions of FBS at 10% in DMEM, and incubating the samples at 37 °C. Results showed that the nanoparticles were stable at least up to 72 h in all investigated media.

4. Determination of PERFECTA limit of Detection (LOD) by RAMAN

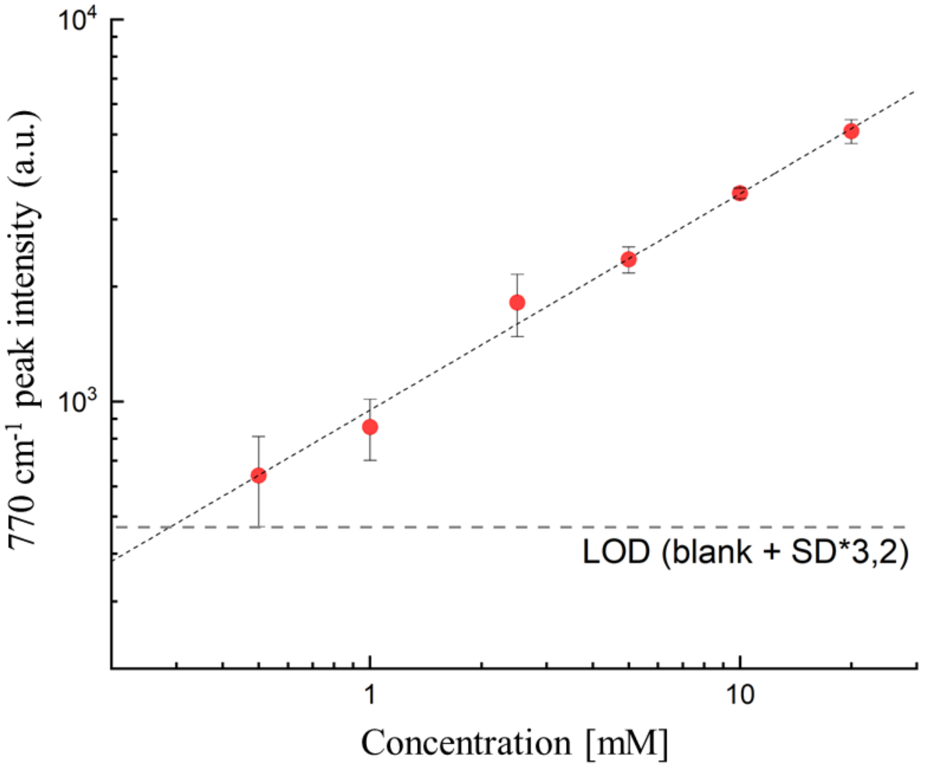

Figure S2. Evaluation of PERFECTA LOD in biological fluids by RAMAN spectroscopy. For Raman microscopy, a limit of detection (LOD) has been estimated in cell culture medium. For this purpose PERFECTA formulation was diluted 1/6 (v/v) in the biological culture medium DMEM supplemented with 10% FBS. The LOD has been estimated as the lowest PERFECTA concentration likely to be reliably distinguished from the blank sample (medium + FBS). LOD is calculated as the (mean value + 3.2 SD) obtained from replicates of measurements of the blank sample. Results showed that for PERFECTA this value is equal to 0.3 mM.

5. Simulation of Raman Spectra by DFT calculations

5.1. Equilibrium structures of the fluorinated probes

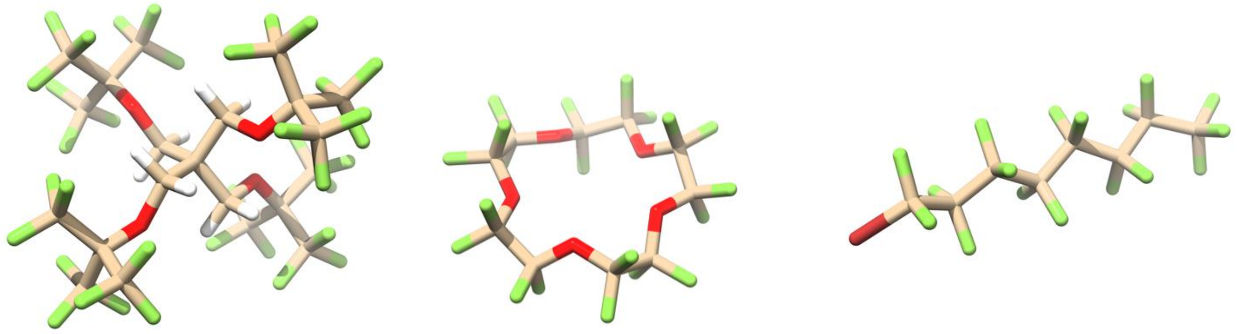

PERFECTA PFCE PFOB

Figure S3. Determination of the equilibrium structures of the fluorinated moleculesby DFT calculations.

The Raman spectra of the three fluorinated probes here considered have been simulated in the region of interest for the selected markers by a weighted sum of Lorenztian functions (FWHM = 10 cm^{-1}) centered at the wavenumbers computed by DFT; the weights of the Lorenztian functions have been fixed at the values of the computed Raman activities (units of $Å^4$/amu). For DFT calculations, the assessment of the lowest energy conformations of PFCE and PFOB was carried out with the help of the Avogadro software (version 1.2.0)[3] and the MMFF94 molecular mechanics force field[4].

Based on the inspection of the nuclear displacements reported in Figure S3, and the related analysis of the displacements in terms of internal coordinates (Table S2) that follows Wilson's approach[5] and the implementation described in a previous work[6], we propose the following assignment of the selected markers of the three fluorinated probes:

PERFECTA (763 cm^{-1}): collective symmetric stretching (inner CC stretching, out-of-phase with respect to CF and CC stretching of the CF_3 units; minor contribution from CO stretching); collective symmetric bending (CCF, FCF; umbrella-like)

PFCE (820 cm^{-1}): collective COC bending; minor contributions from collective (CO, CF) stretching

PFOB (724 cm^{-1}): collective (CC, CF) stretching; collective CCC bending out-of-phase with respect to FCF bending

5.2. DFT-simulated RAMAN spectra

	expt. Raman (cm-1)	DFT (cm-1)	DFT Raman activity (Å4/amu)
PERFECTA	770	763	33
PFCE	820	818	25
PFOB	724	723	13

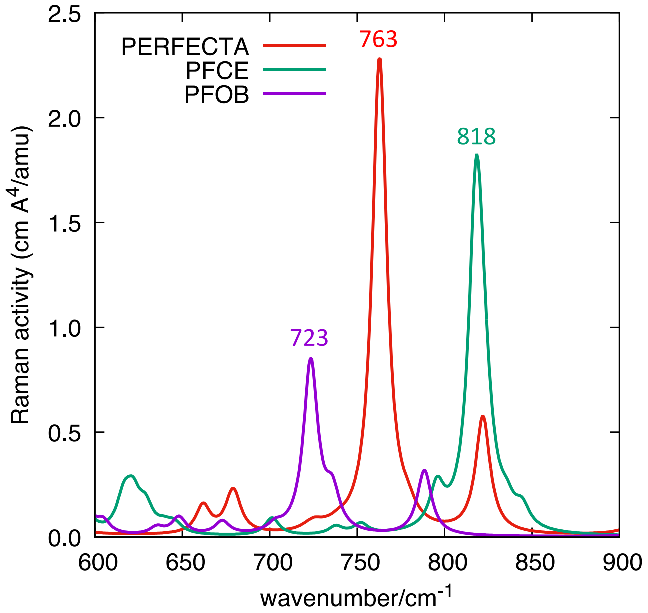

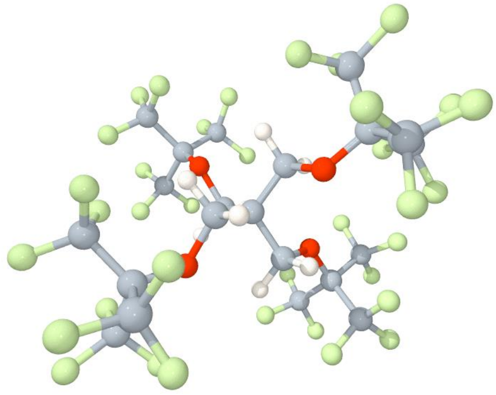

PERFECTA

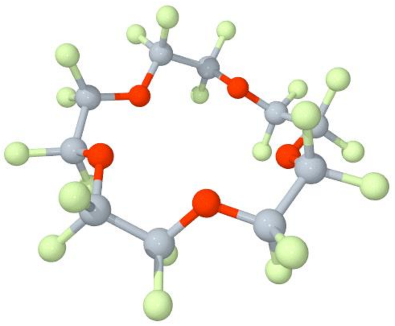

PFCE

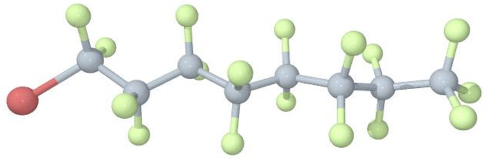

PFOB

Figure S4. DFT-simulated RAMAN spectra and animations of the nuclear displacements associated to the Raman markers.

========

PERFECTA
========

```
 Analysis of the mode #         124  ---   762.60164289037527      [cm**-1]
   MODE #    124    762.60      IR:            0.083    Raman:           32.884
 STR      1    2             C  C        -0.019 ---------------
 STR      1    5             C  C        -0.022 ------------------
 STR      1    8             C  C        -0.021 -----------------
 STR      1   11             C  C        -0.020 ----------------
 STR      2   14             C  O         0.009 +++++++
 STR      5   15             C  O         0.010 ++++++++
 STR      8   16             C  O         0.010 ++++++++
 STR     11   17             C  O         0.011 +++++++++
 STR     14   18             O  C         0.012 ++++++++++
 STR     15   31             O  C         0.013 ++++++++++
 STR     16   44             O  C         0.014 +++++++++++
 STR     17   57             O  C         0.015 ++++++++++++
 STR     18   19             C  C         0.022 ++++++++++++++++++
 STR     18   23             C  C         0.029 ++++++++++++++++++++++++
 STR     18   27             C  C         0.028 +++++++++++++++++++++++
 STR     19   20             C  F         0.018 +++++++++++++++
 STR     19   21             C  F         0.020 ++++++++++++++++
 STR     19   22             C  F         0.021 +++++++++++++++++
 STR     23   24             C  F         0.021 +++++++++++++++++
 STR     23   25             C  F         0.024 +++++++++++++++++++
 STR     23   26             C  F         0.023 +++++++++++++++++++
 STR     27   28             C  F         0.024 ++++++++++++++++++++
 STR     27   29             C  F         0.021 +++++++++++++++++
 STR     27   30             C  F         0.020 ++++++++++++++++
 STR     31   32             C  C         0.023 +++++++++++++++++++
 STR     31   36             C  C         0.030 ++++++++++++++++++++++++
 STR     31   40             C  C         0.031 +++++++++++++++++++++++++
 STR     32   33             C  F         0.021 +++++++++++++++++
 STR     32   34             C  F         0.019 +++++++++++++++
 STR     32   35             C  F         0.022 ++++++++++++++++++
 STR     36   37             C  F         0.022 ++++++++++++++++++
 STR     36   38             C  F         0.025 ++++++++++++++++++++
 STR     36   39             C  F         0.022 ++++++++++++++++++
 STR     40   41             C  F         0.025 +++++++++++++++++++++
 STR     40   42             C  F         0.025 ++++++++++++++++++++
 STR     40   43             C  F         0.021 +++++++++++++++++
 STR     44   45             C  C         0.031 +++++++++++++++++++++++++
 STR     44   49             C  C         0.025 ++++++++++++++++++++
 STR     44   53             C  C         0.033 ++++++++++++++++++++++++++++
 STR     45   46             C  F         0.024 ++++++++++++++++++++
 STR     45   47             C  F         0.023 +++++++++++++++++++
 STR     45   48             C  F         0.026 +++++++++++++++++++++
 STR     49   50             C  F         0.020 ++++++++++++++++
 STR     49   51             C  F         0.024 ++++++++++++++++++++
 STR     49   52             C  F         0.022 ++++++++++++++++++
 STR     53   54             C  F         0.027 ++++++++++++++++++++++
 STR     53   55             C  F         0.023 +++++++++++++++++++
 STR     53   56             C  F         0.026 ++++++++++++++++++++++
 STR     57   58             C  C         0.025 ++++++++++++++++++++
 STR     57   62             C  C         0.034 ++++++++++++++++++++++++++++
 STR     57   66             C  C         0.032 ++++++++++++++++++++++++++
 STR     58   59             C  F         0.020 +++++++++++++++++
 STR     58   60             C  F         0.022 ++++++++++++++++++
 STR     58   61             C  F         0.024 ++++++++++++++++++++
 STR     62   63             C  F         0.024 +++++++++++++++++++
 STR     62   64             C  F         0.028 +++++++++++++++++++++++
 STR     62   65             C  F         0.027 ++++++++++++++++++++++
```

STR	66	67		C	F		0.026	+++++++++++++++++++++
STR	66	68		C	F		0.025	++++++++++++++++++++
STR	66	69		C	F		0.023	+++++++++++++++++++
BEND	2	1	5	C	C	C	-0.004	---
BEND	2	1	8	C	C	C	0.007	+++++
BEND	5	1	8	C	C	C	-0.004	---
BEND	5	1	11	C	C	C	0.006	++++
BEND	1	2	14	C	C	O	-0.008	------
BEND	3	2	4	H	C	H	0.004	+++
BEND	1	5	7	C	C	H	0.005	+++
BEND	1	5	15	C	C	O	-0.007	-----
BEND	6	5	7	H	C	H	0.004	+++
BEND	1	8	9	C	C	H	0.007	+++++
BEND	1	8	16	C	C	O	-0.010	--------
BEND	1	11	13	C	C	H	0.006	+++++
BEND	1	11	17	C	C	O	-0.011	---------
BEND	12	11	13	H	C	H	0.004	+++
BEND	2	14	18	C	O	C	-0.004	---
BEND	8	16	44	C	O	C	-0.006	-----
BEND	11	17	57	C	O	C	-0.008	------
BEND	14	18	23	O	C	C	0.005	++++
BEND	14	18	27	O	C	C	0.004	+++
BEND	23	18	27	C	C	C	-0.006	----
BEND	18	19	20	C	C	F	-0.028	----------------------
BEND	18	19	21	C	C	F	-0.025	--------------------
BEND	18	19	22	C	C	F	-0.020	----------------
BEND	20	19	21	F	C	F	0.025	+++++++++++++++++++++
BEND	20	19	22	F	C	F	0.026	++++++++++++++++++++++
BEND	21	19	22	F	C	F	0.025	++++++++++++++++++++
BEND	18	23	24	C	C	F	-0.031	------------------------
BEND	18	23	25	C	C	F	-0.023	------------------
BEND	18	23	26	C	C	F	-0.027	---------------------
BEND	24	23	25	F	C	F	0.029	++++++++++++++++++++++++
BEND	24	23	26	F	C	F	0.027	++++++++++++++++++++++
BEND	25	23	26	F	C	F	0.029	++++++++++++++++++++++++
BEND	18	27	28	C	C	F	-0.022	-----------------
BEND	18	27	29	C	C	F	-0.027	----------------------
BEND	18	27	30	C	C	F	-0.029	-----------------------
BEND	28	27	29	F	C	F	0.027	++++++++++++++++++++++
BEND	28	27	30	F	C	F	0.028	+++++++++++++++++++++++
BEND	29	27	30	F	C	F	0.027	+++++++++++++++++++++++
BEND	15	31	36	O	C	C	0.004	+++
BEND	15	31	40	O	C	C	0.005	++++
BEND	36	31	40	C	C	C	-0.005	----
BEND	31	32	33	C	C	F	-0.027	---------------------
BEND	31	32	34	C	C	F	-0.028	----------------------
BEND	31	32	35	C	C	F	-0.021	-----------------
BEND	33	32	34	F	C	F	0.026	++++++++++++++++++++++
BEND	33	32	35	F	C	F	0.026	+++++++++++++++++++++
BEND	34	32	35	F	C	F	0.028	+++++++++++++++++++++++
BEND	31	36	37	C	C	F	-0.030	------------------------
BEND	31	36	38	C	C	F	-0.023	------------------
BEND	31	36	39	C	C	F	-0.029	-----------------------
BEND	37	36	38	F	C	F	0.029	++++++++++++++++++++++++
BEND	37	36	39	F	C	F	0.029	++++++++++++++++++++++++
BEND	38	36	39	F	C	F	0.028	+++++++++++++++++++++++
BEND	31	40	41	C	C	F	-0.026	--------------------
BEND	31	40	42	C	C	F	-0.029	-----------------------
BEND	31	40	43	C	C	F	-0.032	--------------------------
BEND	41	40	42	F	C	F	0.030	++++++++++++++++++++++++
BEND	41	40	43	F	C	F	0.032	++++++++++++++++++++++++++
BEND	42	40	43	F	C	F	0.030	+++++++++++++++++++++++++
BEND	16	44	53	O	C	C	0.007	++++++
BEND	45	44	53	C	C	C	-0.007	-----

```
BEND   44  45  46        C  C  F      -0.031 ------------------------
BEND   44  45  47        C  C  F      -0.032 -------------------------
BEND   44  45  48        C  C  F      -0.024 -------------------
BEND   46  45  47        F  C  F       0.029 +++++++++++++++++++++++
BEND   46  45  48        F  C  F       0.030 ++++++++++++++++++++++++
BEND   47  45  48        F  C  F       0.031 +++++++++++++++++++++++++
BEND   44  49  50        C  C  F      -0.031 -------------------------
BEND   44  49  51        C  C  F      -0.022 -----------------
BEND   44  49  52        C  C  F      -0.029 -----------------------
BEND   50  49  51        F  C  F       0.029 +++++++++++++++++++++++
BEND   50  49  52        F  C  F       0.029 +++++++++++++++++++++++
BEND   51  49  52        F  C  F       0.028 ++++++++++++++++++++++
BEND   44  53  54        C  C  F      -0.027 ----------------------
BEND   44  53  55        C  C  F      -0.035 ----------------------------
BEND   44  53  56        C  C  F      -0.030 ------------------------
BEND   54  53  55        F  C  F       0.034 +++++++++++++++++++++++++++
BEND   54  53  56        F  C  F       0.032 +++++++++++++++++++++++++
BEND   55  53  56        F  C  F       0.031 +++++++++++++++++++++++++
BEND   17  57  62        O  C  C       0.007 ++++++
BEND   62  57  66        C  C  C      -0.007 -----
BEND   57  58  59        C  C  F      -0.032 -------------------------
BEND   57  58  60        C  C  F      -0.029 -----------------------
BEND   57  58  61        C  C  F      -0.023 -------------------
BEND   59  58  60        F  C  F       0.029 +++++++++++++++++++++++
BEND   59  58  61        F  C  F       0.029 +++++++++++++++++++++++
BEND   60  58  61        F  C  F       0.029 +++++++++++++++++++++++
BEND   57  62  63        C  C  F      -0.036 ----------------------------
BEND   57  62  64        C  C  F      -0.028 ----------------------
BEND   57  62  65        C  C  F      -0.031 ------------------------
BEND   63  62  64        F  C  F       0.034 +++++++++++++++++++++++++++
BEND   63  62  65        F  C  F       0.032 +++++++++++++++++++++++++
BEND   64  62  65        F  C  F       0.033 ++++++++++++++++++++++++++
BEND   57  66  67        C  C  F      -0.024 --------------------
BEND   57  66  68        C  C  F      -0.031 ------------------------
BEND   57  66  69        C  C  F      -0.032 --------------------------
BEND   67  66  68        F  C  F       0.031 ++++++++++++++++++++++++
BEND   67  66  69        F  C  F       0.032 +++++++++++++++++++++++++
BEND   68  66  69        F  C  F       0.030 ++++++++++++++++++++++++
TORS    1   2          C  C        0.014 ++++++++++++
TORS    1   5          C  C       -0.010 --------
TORS    1   8          C  C        0.027 +++++++++++++++++++++
TORS    1  11          C  C       -0.026 ---------------------
TORS    2  14          C  O       -0.004 ---
TORS    5  15          C  O        0.007 ++++++
TORS    8  16          C  O       -0.010 --------
TORS   11   17          C  O        0.010 +++++++
TORS   15   31          O  C       -0.004 ---
TORS   18   27          C  C        0.005 +++
TORS   31   36          C  C        0.005 +++
TORS   31   40          C  C       -0.004 ---
TORS   44   45          C  C       -0.005 ----
TORS   57   66          C  C        0.006 ++++
====
PFCE
====
Analysis of the mode #        71  ---   818.00001983195000      [cm**-1]
  MODE #    71    818.00      IR:            3.847    Raman:           24.539
STR     1   6           O  C        0.021 +++
STR     1  15           O  C        0.034 +++++
STR     2  14           O  C        0.027 ++++
STR     3  11           O  C        0.045 ++++++
STR     3  12           O  C        0.026 ++++
STR     4   7           O  C        0.076 +++++++++++
STR     4   8           O  C        0.078 ++++++++++++
```

```
STR      5    9              O  C          0.043 ++++++
STR      5   10              O  C          0.027 ++++
STR      6   17              C  F          0.019 +++
STR      7   19              C  F          0.062 +++++++++
STR      8   20              C  F          0.069 ++++++++++
STR      8   21              C  F          0.052 +++++++
STR     10   24              C  F          0.023 +++
STR     10   25              C  F          0.028 ++++
STR     11   26              C  F          0.020 +++
STR     11   27              C  F          0.037 +++++
STR     14   32              C  F          0.022 +++
STR     14   33              C  F          0.023 +++
STR     15   34              C  F          0.020 +++
STR     15   35              C  F          0.028 ++++
BEND    6    1   15          C  O  C      -0.059 ---------
BEND   13    2   14          C  O  C      -0.050 -------
BEND   11    3   12          C  O  C      -0.089 -------------
BEND    7    4    8          C  O  C      -0.194 ----------------------------
BEND    9    5   10          C  O  C      -0.061 ---------
BEND    1    6    7          O  C  C      -0.026 ---
BEND    4    7    6          O  C  C      -0.097 --------------
BEND    4    7   19          O  C  F       0.071 ++++++++++
BEND   18    7   19          F  C  F       0.027 ++++
BEND    4    8    9          O  C  C      -0.091 -------------
BEND    4    8   20          O  C  F       0.081 ++++++++++++
BEND    4    8   21          O  C  F       0.049 +++++++
BEND    9    8   20          C  C  F      -0.044 ------
BEND    9    8   21          C  C  F      -0.021 ---
BEND   20    8   21          F  C  F       0.022 +++
BEND    5    9    8          O  C  C      -0.025 ---
BEND    5    9   23          O  C  F      -0.033 -----
BEND    5   10   11          O  C  C      -0.034 -----
BEND    3   11   10          O  C  C      -0.041 ------
BEND    3   11   26          O  C  F       0.025 +++
BEND    3   11   27          O  C  F       0.037 +++++
BEND   10   11   26          C  C  F      -0.020 ---
BEND    3   12   13          O  C  C      -0.046 -------
BEND    3   12   29          O  C  F       0.027 ++++
BEND    2   13   12          O  C  C      -0.028 ----
BEND    2   14   15          O  C  C      -0.022 ---
BEND    2   14   32          O  C  F       0.022 +++
BEND    1   15   14          O  C  C      -0.031 ----
BEND    1   15   35          O  C  F       0.021 +++
TORS    6    7               C  C         -0.029 ----
TORS    8    9               C  C         -0.040 ------
```

```
===
PFOB
====

 Analysis of the mode #          54  ---   723.39534724462442       [cm**-1]
   MODE #    54    723.40       IR:             0.312    Raman:            12.959
 STR     1    9              C  F         0.014 ++++
 STR     2    3              C  C        -0.016 -----
 STR     2   12              C  F        -0.011 ---
 STR     2   13              C  F        -0.020 ------
 STR     3    4              C  C        -0.033 -----------
 STR     3   14              C  F        -0.027 ---------
 STR     3   15              C  F        -0.037 ------------
 STR     4    5              C  C        -0.045 ---------------
 STR     4   16              C  F        -0.040 -------------
 STR     4   17              C  F        -0.047 ----------------
 STR     5    6              C  C        -0.049 ----------------
 STR     5   18              C  F        -0.047 ---------------
 STR     5   19              C  F        -0.050 -----------------
 STR     6    7              C  C        -0.046 ---------------
 STR     6   20              C  F        -0.047 ----------------
 STR     6   21              C  F        -0.045 ---------------
 STR     7    8              C  C        -0.031 ----------
 STR     7   22              C  F        -0.033 -----------
 STR     7   23              C  F        -0.040 -------------
 STR     8   24              C Br         -0.012 ----
 STR     8   25              C  F        -0.017 -----
 STR     8   26              C  F        -0.021 -------
 BEND    2    1    9          C  C  F      -0.010 ---
 BEND    2    1   10          C  C  F      -0.020 ------
 BEND    2    1   11          C  C  F      -0.023 -------
 BEND    9    1   10          F  C  F       0.021 +++++++
 BEND    9    1   11          F  C  F       0.019 ++++++
 BEND   10    1   11          F  C  F       0.015 +++++
 BEND    1    2    3          C  C  C       0.031 ++++++++++
 BEND    1    2   13          C  C  F       0.009 +++
 BEND    3    2   13          C  C  F      -0.019 ------
 BEND   12    2   13          F  C  F      -0.023 -------
 BEND    2    3    4          C  C  C       0.063 +++++++++++++++++++++
 BEND    4    3   15          C  C  F      -0.014 ----
 BEND   14    3   15          F  C  F      -0.052 -----------------
 BEND    3    4    5          C  C  C       0.082 +++++++++++++++++++++++++++
 BEND    3    4   16          C  C  F      -0.013 ----
 BEND    5    4   16          C  C  F       0.010 +++
 BEND   16    4   17          F  C  F      -0.071 ------------------------
 BEND    4    5    6          C  C  C       0.088 +++++++++++++++++++++++++++++
 BEND    4    5   18          C  C  F      -0.017 -----
 BEND    6    5   18          C  C  F       0.013 ++++
 BEND   18    5   19          F  C  F      -0.078 --------------------------
 BEND    5    6    7          C  C  C       0.081 +++++++++++++++++++++++++++
 BEND    5    6   20          C  C  F      -0.018 -----
 BEND    7    6   20          C  C  F       0.011 +++
 BEND   20    6   21          F  C  F      -0.075 -------------------------
 BEND    6    7    8          C  C  C       0.065 ++++++++++++++++++++++
 BEND    6    7   23          C  C  F      -0.014 ----
 BEND   22    7   23          F  C  F      -0.060 --------------------
 BEND    7    8   24          C  C Br       0.036 ++++++++++++
 BEND    7    8   26          C  C  F      -0.011 ---
 BEND   25    8   26          F  C  F      -0.035 -----------
```

Table S2. Output of the analysis of the normal modes related to the selected Raman markers. For each case, the output lists the relevant contributions from valence internal coordinates to the nuclear displacement of the normal mode. The atom numbering scheme is provided in the **Figure S5** below.

5.3. DFT optimized molecular structures

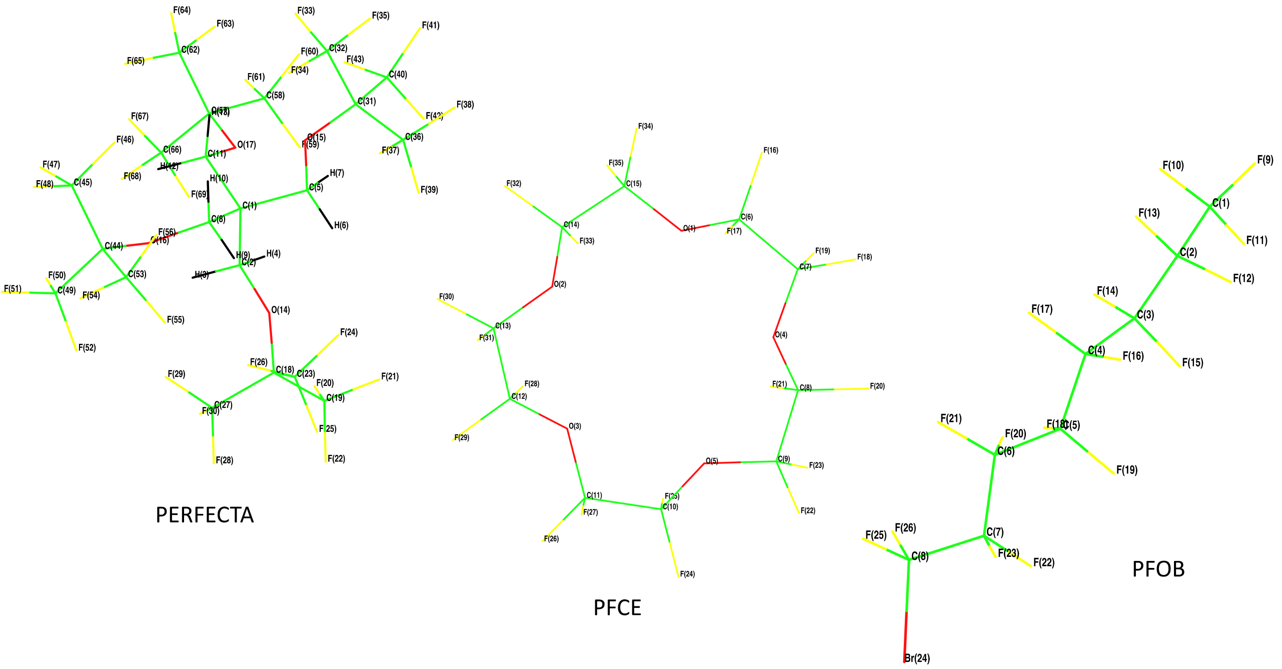

Figure S5. Atom numbering scheme for the DFT calculations of the three fluorinated probes.

6. Fluorescence imaging studies

6.1. Confocal microscopy on labeled cells

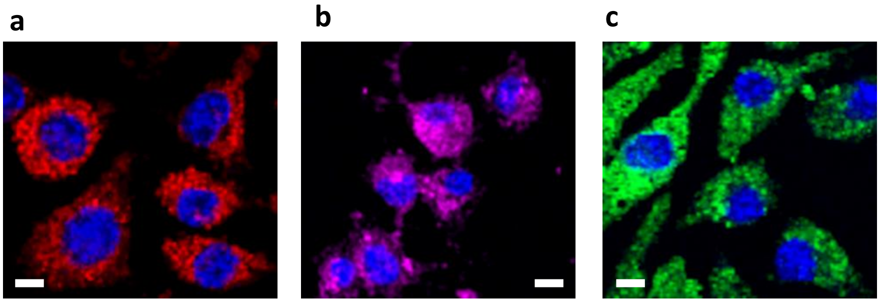

Figure S6. Detection of labelled murine microglial cells (BV-2) by confocal microscopy. Cells were incubated with each set of fluorescent formulation at the same concentration of fluorine (**a:** PERFECTA, **b:** PFOB and **c:** PFCE). Labelled cells were then visualised by fluorescence imaging. Scale bar: 5 µm.

6.2. Confocal microscopy on tissue sections

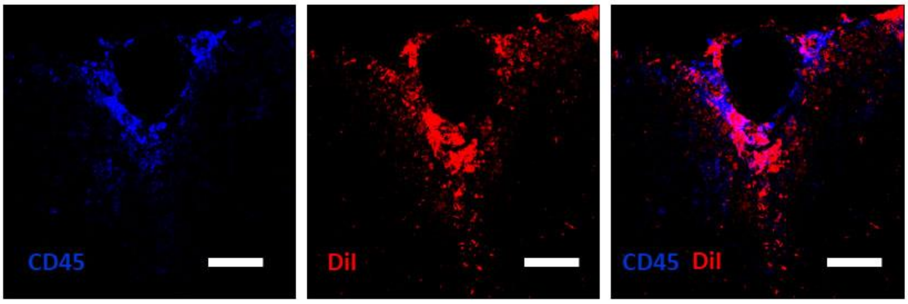

Figure S7. Confocal microscopy of tissue section collected from spinal cord of an EAE mice. Fluorescence imaging performed in the cervical spinal cord section of a pathological mice, indicating the localization of the leukocyte-rich region (CD45 staining, blue) compared to PERFECTA (Dil label, red). Scale bar: 50 µm.

7. From *in-vivo* MRI to tissue RAMAN microscopy

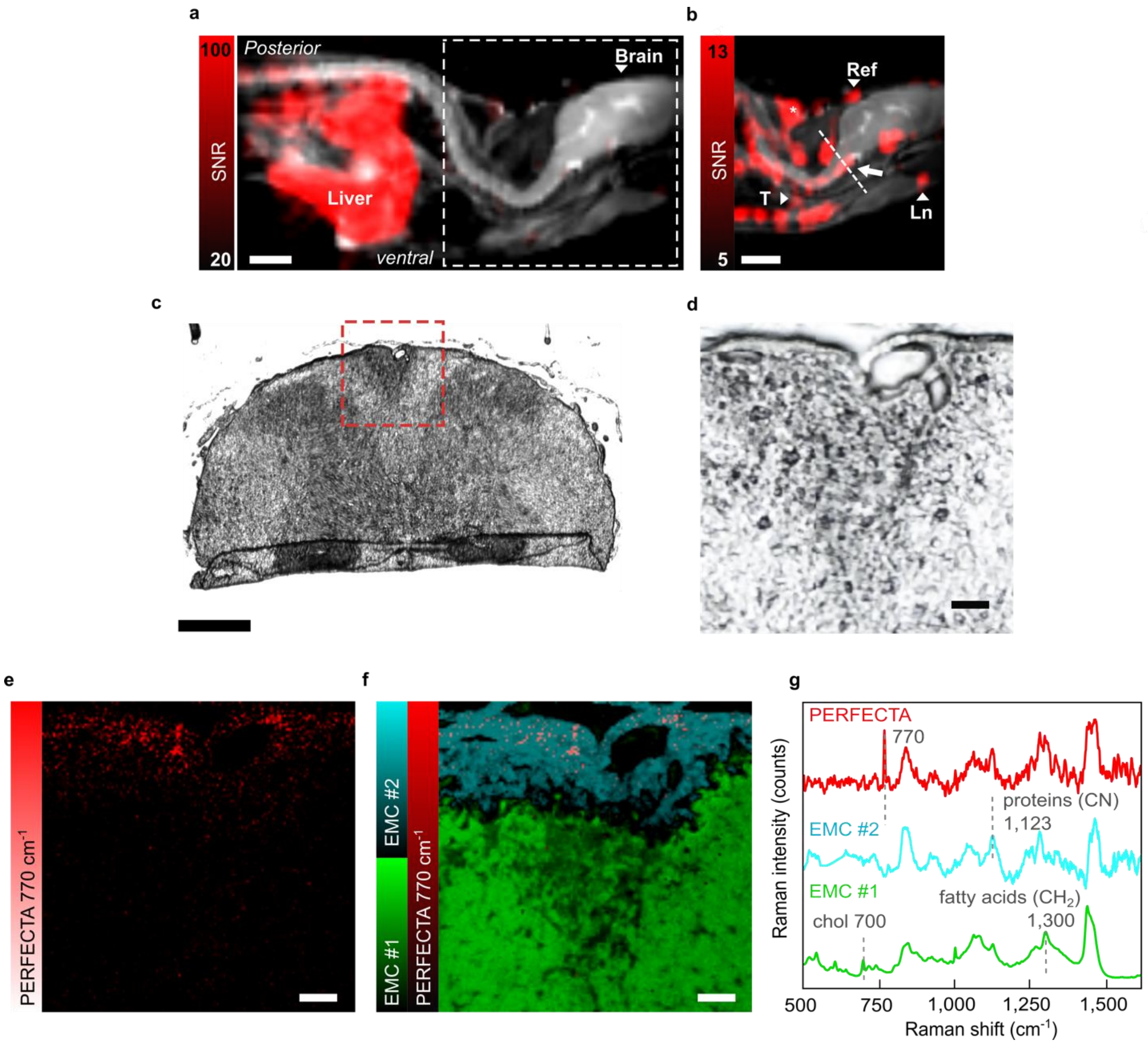

Figure S8. Representative biological replicate of data shown in Figure 5. a. *In-vivo* ^{19}F-MRI at the specific resonance frequency of PERFECTA (red colour scale) acquired over the mouse body and merged with the anatomical image (T, thymus and Ln, cervical lymph nodes; ref, external reference of PERFECTA). Scale bar, 5 mm. b. Distinct red spots were clearly detected along the spinal cord (white arrow) where Raman imaging was successively performed. Scale bar, 5 mm. c. Bright field (BF) image of the unstained fresh-frozen tissue section collected from mice spinal cord (cervical region) and studied by Raman imaging (red dashed square). Scale bar, 0.5 mm. d. BF image of the selected tissue regions. e. Raman map of PERFECTA band. f. Merge of Raman maps related to EMC#1, 2 and to PERFECTA. g. typical (averaged) Raman spectra obtained by ex-vivo Raman imaging of tissue slice. Empty Modelling Component (EMC) #1 mostly represents white matter (green) (chol: cholesterol); EMC #2 mostly represents epithelium and immune cell infiltration (cyan); PERFECTA spectrum is the average of image pixels reporting signal at 770 cm^{-1}. Raman maps on tissue slices were collected in raster scan modality using a step size of 2.5 µm and a single 2 s acquisition for each step. For d and e, scale bar = 50 µm

8. Supplementary references

(1) Tirotta, I.; Mastropietro, A.; Cordiglieri, C.; Gazzera, L.; Baggi, F.; Baselli, G.; Grazia Bruzzone, M.; Zucca, I.; Cavallo, G.; Terraneo, G.; Baldelli Bombelli, F.; Metrangolo, P.; Resnati, G. A Superfluorinated Molecular Probe for Highly Sensitive in Vivo 19F-MRI. *J. Am. Chem. Soc.* **2014**, *136* (24), 8524–8527.

(2) Chirizzi, C.; De Battista, D.; Tirotta, I.; Metrangolo, P.; Comi, G.; Bombelli, F. B.; Chaabane, L. Multispectral MRI with Dual Fluorinated Probes to Track Mononuclear Cell Activity in Mice. *Radiology* **2019**, *291* (2), 351–357.

(3) Hanwell, M. D.; Curtis, D. E.; Lonie, D. C.; Vandermeersch, T.; Zurek, E.; Hutchison, G. R. Avogadro: An Advanced Semantic Chemical Editor, Visualization, and Analysis Platform. *J. Cheminform.* **2012**, *4* (1), 17.

(4) Halgren, T. A. Merck Molecular Force Field. I. Basis, Form, Scope, Parameterization, and Performance of MMFF94. *J. Comput. Chem.* **1996**, *17* (5–6), 490–519.

(5) Wilson, E. B.; Decius, J. C.; Cross, P. C.; Sundheim, B. R. Molecular Vibrations: The Theory of Infrared and Raman Vibrational Spectra. *J. Electrochem. Soc.* **1955**, *102* (9), 235C.

(6) Radice, S.; Tommasini, M.; Castiglioni, C. Two Dimensional Correlation Raman Spectroscopy of Perfluoropolyethers: Effect of Peroxide Groups. *J. Mol. Struct.* **2010**, *974* (1–3), 73–79.
